# Facile and Green Preparation of Pectin/Cellulose Composite Films with Enhanced Antibacterial and Antioxidant Behaviors

**DOI:** 10.3390/polym11010057

**Published:** 2019-01-02

**Authors:** Shan Ye, Zhongjie Zhu, Yanyi Wen, Chen Su, Lei Jiang, Shu He, Wei Shao

**Affiliations:** 1 Jiangsu Co-Innovation Center of Efficient Processing and Utilization of Forest Resources, Nanjing Forestry University, Nanjing 210037, China; 13451867328@163.com; 2 Jiangsu Key Lab for the Chemistry and Utilization of Agricultural and Forest Biomass, Nanjing Forestry University, Nanjing 210037, China; 3 College of Chemical Engineering, Nanjing Forestry University, Nanjing 210037, China; 13382367651@163.com (Z.Z.); wenyanyis@163.com (Y.W.); 13260866882@163.com (C.S.); 15655679696@163.com (L.J.); heshu999@163.com (S.H.)

**Keywords:** cellulose, pectin, antibacterial, antioxidant

## Abstract

Novel bioactive films based on pectin and cellulose (PC) with different loadings of tea polyphenols and cinnamaldehyde were successfully prepared. A thermal stability was tested, and the results showed that the thermal stability decreased slightly after loading with cinnamaldehyde and tea polyphenols, compared to PC films. The antimicrobial and antioxidant capacities were also investigated. Results showed that PC composite films had good DPPH radical and hydroxyl radical scavenging activities and excellent antibacterial activities against *Escherichia coli*, *Candida albicans* and *Staphylococcus aureus.* Based on the results, the great antioxidant and antibacterial activities of the tea polyphenol and cinnamaldehyde loaded PC films make them suitable for food packaging and preservation.

## 1. Introduction

Food packaging materials need to possess good mechanical, air, and moisture barrier properties and be nontoxic. The food packages sold in the market nowadays are petroleum-based plastics, which are non-biodegradable, directly affecting the marine environment and physical security. Moreover, they do not supply any antibacterial or antioxidant performance [1]. Therefore, it is crucial to develop novel food packaging materials that are biodegradable with good antibacterial, antioxidant, and barrier properties, to extend shelf-life and improve food storage effects with environment-friendly activities [2]. Thus, novel films providing green and sustainable packaging activities are relatively attractive [3].

Pectin is a family of polysaccharides and oligosaccharides with diverse structures which is located in the cell wall of most plants, such as apple and citrus [4]. It has a linear structure that is rich in galacturonic acid. The molecular weight of pectin is in the range of 50,000–150,000 Da. Pectin has many different applications depending on its degree of esterification and polymerization [5]. Owing to its bioactivity, biocompatibility, biodegradability, renewability, low cost, and easy modification, pectin is becoming a potential candidate in many fields, such as medical products, cosmetics, bioimplants, drug delivery, tissue engineering, herbicides, and the textile and food industries. Pectin films used in the food industry exhibit good oxygen barrier properties with good hardness and adhesiveness. Despite their advantages, pectin films also present some limitations, such as rigidity and brittleness, and poor water barrier properties due to their high water sensitivity [6]. Therefore, improvements to pectin films should be made [7].

Pectin has a strong hydrophilic property, so it needs to be cross-linked in order to improve the physical properties and its stability in water. Chemical cross-linking and physical cross-linking are commonly used methods. Chemical cross-linking is a versatile method, forming highly stable and strong films via the formation of covalent bonds. The most commonly used cross-linkers are divalent ions (Ca^2+^, Mg^2+^), glutaraldehyde, diimines, and so on. Meanwhile, the films formed by physical cross-linking methods are less stable due to the formation of secondary and reversible bonds [8]. However, they are favored for the encapsulation of labile bioactive substances, antibacterial agents, antioxidants, anti-browning agents, and flavors and colorants inside hydrogels, especially if the formation occurs under mild conditions in the absence of organic solvents [9,10].

It was reported that blending pectin with other polymers could improve the performance of pectin-based materials in the fields of food applications, drug delivery, and tissue engineering [7]. Aloe vera and curcumin loaded oxidized pectin-gelatin matrices as composite wound care devices exerted a strong anti-inflammatory effect and prominent scar prevention, making the biocomposite dressings effective in wound management [11]. Pectin/sodium alginate composite films cross-linked with zinc ions were prepared by casting methods, and exhibited antimicrobial activity against *Staphylococcus aureus*, *Escherichia coli*, and *Candida albicans*, which suggested that pectin-based films can be exploitable as novel bioactive biomaterials for use in medical devices [12]. Whey protein/pectin films prepared in the presence of transglutaminase successfully prevented microbial growth [13]. The highly methoxylated pectin and poly(ethylene glycol) films prepared by the solution casting method resulted in interesting bio-inspired edible films, showing potential to compete with commercially used synthetic package materials [14].

Cellulose is the most abundant, renewable, and environment-friendly polysaccharide, consisting of a linear chain of β-1-4-linked D-glucopyranose repeating units with a large amount of hydroxyl groups. It has wide applications for packaging materials in the paper, medicine, cosmetic, food, and biomedical industries, since it has low cost, good biodegradability, biocompatibility, nontoxicity, chemical and thermal stability, and high mechanical strength [15].

In this study, we prepared pectin/cellulose (PC) composite films by a facile method. Furthermore, we incorporated natural antioxidant tea polyphenols (TP) and the natural antibacterial agent cinnamaldehyde into the prepared PC films, to endow them with antioxidant and antibacterial properties. To the best of our knowledge, the combination of TP and cinnamaldehyde into PC films has not previously been reported.

## 2. Materials and Methods

### 2.1. Preparation of PC Composite Films

**Figure 1:**
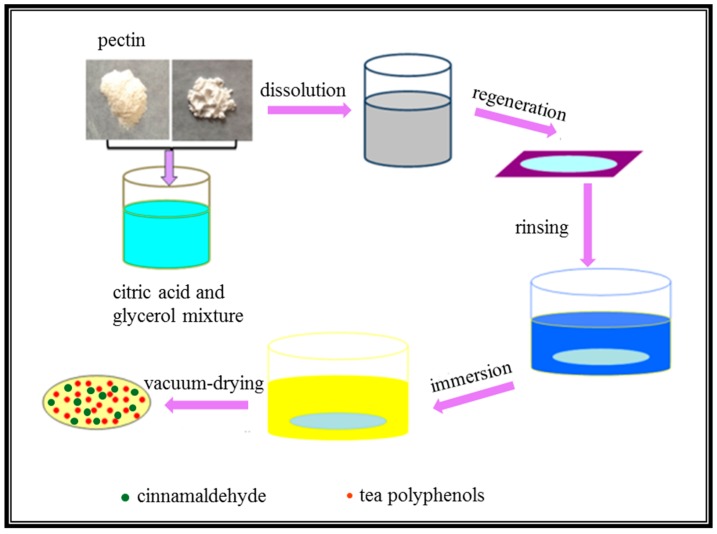
Schematic illustration of the preparation of pectin/cellulose (PC) composite films.

Pectin was added into a mixture of 8% citric acid and 30% glycerol, and stirred at 70 °C for 2 h to achieve a concentration of 4%. Cellulose solution with 6% concentration was prepared by dissolving microcrystalline cellulose powder into pectin solution and stirring at 70 °C for 4 h. Then, the cellulose/pectin mixture was casted into acrylic plates with diameters of 90 mm at 4 °C for 30 min, and soaked in a 1.5% epichlorohydrin ethanol solution for 6 h, followed by rinsing with de-ionized water. Then, the formed hydrogels were immersed into TP solutions with concentrations of 0.2, 0.4, 0.6, 0.8, and 1.0 mg/mL for 24 h. The TP-loaded hydrogels were obtained by rinsing with de-ionized water, followed by immersing into cinnamaldehyde ethanol solutions with 1%, 2%, 3%, 4%, and 5% concentration for another 24 h. The obtained hydrogels were rinsed with de-ionized water, followed by vacuum-drying at 50 °C for 12 h. The final films were named as PC_1_, PC_2_, PC_3_, PC_4_, and PC_5_, respectively. The detailed preparation method is listed in Figure 1. The initial TP and cinnamaldehyde concentrations are listed in Table 1.

### 2.2. Characterization

A JSM-7600F Scanning Electron Microscope (SEM) operating at an accelerating voltage of 10–15 kV was used to investigate the surface morphologies of the PC and PC_5_ composite membranes. The samples were coated with a thin layer of platinum under high-vacuum conditions (20 mA, 100 s). FTIR spectra were recorded on a Spectrum Two Spectrometer (Perkin Elmer, Akron, OH, USA) with a wavenumber range of 4000–400 cm^−1^ at a resolution of 4 cm^−1^. Thermogravimetric (TG) analysis was carried out by using a TA Instruments model Q5000 TGA. The samples were heated from 25 to 600 °C with a heating rate of 10 °C/min under nitrogen atmosphere.

### 2.3. Antibacterial and Antifungal Activities

The antibacterial and antifungal activities of the PC composite films were investigated by the disk diffusion method against *E. coli*, *C. albicans*, and *S. aureus*. PC and PC composite films were cut into round shapes with 10 mm diameters, and sterilized by an ultraviolet lamp for 60 min. Lawns of test bacteria (about 1 × 10^5^ CFU/plate) were prepared on Tryptone Soya Agar plates. The sterilized films were then carefully placed upon the lawns and placed in a 37 °C incubator for 24 h. Then, the inhibitory action of the tested samples on the growth of the bacteria was determined by measuring the diameters of inhibition zones.

### 2.4. Antioxidant Activities

The DPPH (2,2′-diphenyl-1-picrylhydrazyl) radical scavenging activities were determined according to the method of Lee et al. [16]. DPPH was dissolved in ethanol to obtain a concentration of 0.1 mM. PC and PC composite films with diameters of 10 mm were added to 3 mL of DPPH solution and kept in the dark for 30 min at room temperature. Then, the absorbance values of the solution before (*A*_0_) and after (*A*_1_) treatment with PC-based films were measured at λ = 517 nm. The absorbance of ethanol treated with PC based films (*A*_2_) for 30 min at room temperature without any DPPH was also measured. The DPPH radical scavenging percentage was calculated as follows:

$$\mathrm{DPPH}\;\mathrm{radical}\;\mathrm{scavenging}\;\mathrm{percentage}\;(\%)=(1-\frac{A_{1}-A_{2}}{A_{0}})\times 100\%$$

The capacities of PC and PC composite films to scavenge hydroxyl radicals were measured [16]. Briefly, the reaction systems contained phosphate buffer (1.09 mL, 0.2 M, pH 7.4), 1,10-phenanthroline (0.82 mL, 0.5 mM), ferrous sulfate (0.54 mL, 0.75 mM), and PC-based films in a 10 mL beaker. Then, H_2_O_2_ (0.54 mL, 0.01%, *v/v*) was added and incubated at 37 °C for 60 min. The absorbance values before (*A*_0_) and after (*A_S_*) treatment with PC films were measured at λ = 536 nm. De-ionized water was used as the blank, and the absorbance without PC-based films (*A*_b_) was measured at λ = 536 nm. The scavenging percentage of hydroxyl radicals was calculated using the following formula:

$$\mathrm{hydroxyl}\;\mathrm{radical}\;\mathrm{scavenging}\;\mathrm{percentage}\;(\%)=\frac{As-A_{1}}{A_{0}-A_{1}}\times 100\%$$

### 2.5. Mechanical Properties

The tensile properties of PC and PC_5_ films were measured by a dynamic mechanical analyzer (CMT4204, Shenzhen SANS Testing Machine Co., Ltd., Shenzhen, China). Samples were cut manually by a razor blade into strips (50 mm × 5 mm). The static tensile tests were conducted in a ramp displacement mode at an across-head speed of 1 mm/min. Each sample was measured at least five times. The average value and the error bars were calculated.

## 3. Results

### 3.1. SEM Morphology

**Figure 2:**
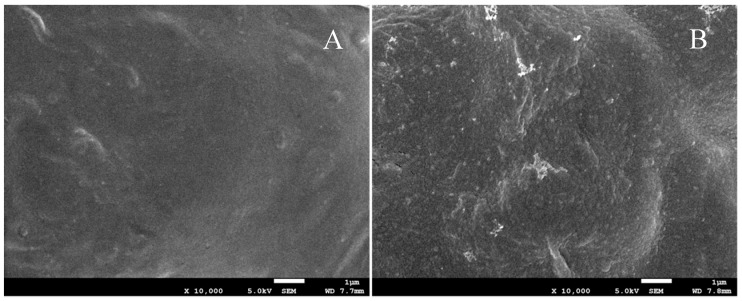
SEM images of (**A**) PC and (**B**) PC_5_ films.

SEM images of PC and PC_5_ films at a magnification of 10,000× are displayed in Figure 2. The surface morphology of PC film (Figure 2A) exhibits a homogenous and smooth surface without any pores. In the case of PC_5_ film, it exhibited a rough surface with the presence of some particles, which is shown in Figure 2B. Clearly, the incorporation of cinnamaldehyde and tea polyphenols to PC film did not significantly change the dense surface morphology except for the increase of roughness. Based on SEM analysis, it is apparent that both the surfaces of PC and PC_5_ films display dense surface morphologies with no presence of pores.

### 3.2. FTIR Characterization

**Figure 3:**
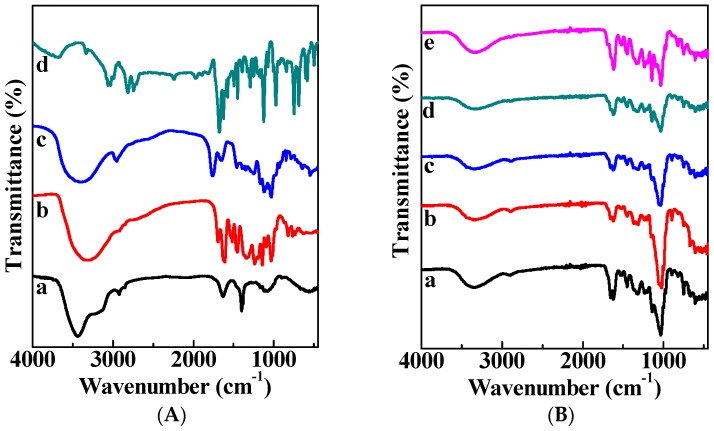
FTIR spectra of PC composite films. (**A**) cellulose (a), tea polyphenols (b), cinnamaldehyde (c), and pectin (d); (**B**) PC_1_ (a), PC_2_ (b), PC_3_ (c), PC_4_ (d), PC_5_ (e).

The FTIR spectrum of cellulose is shown in Figure 3A (Curve a). A broad peak ranging from 3700 to 3200 cm^−1^ is corresponding to the hydroxyl groups and intramolecular hydrogen bond. The peak at 2900 cm^−1^ is attributed to the CH_2_ groups [15]. For Curve b, TP showed typical bands at 1610 and 1510 cm^−1^, which correspond to benzene skeleton C=C vibration. The peak at 1690 cm^−1^ is attributed to C=O stretching [17]. In the case of cinnamaldehyde (Curve c), three characteristic bands at 3027, 1669, and 1624 cm^−1^ are allocated to C–H, C=O, and the aromatic vibrations, respectively, which is in agreement with previous reports on cinnamaldehyde [18]. There are two bands at 1748 and 1626 cm^−1^, attributed to the absorptions by esterified and free carboxyl groups of pectin (Curve d), respectively. For PC composite films (Figure 3B), all the characteristic bands of cellulose, pectin, TP, and cinnamaldehyde can be found in the FTIR spectra of PC_1_ (Curve a), PC_2_ (Curve b), PC_3_ (Curve c), PC_4_ (Curve d), and PC_5_ (Curve e). This confirmed the successful preparation of PC composite films.

### 3.3. Thermogravimetric Analysis

**Figure 4:**
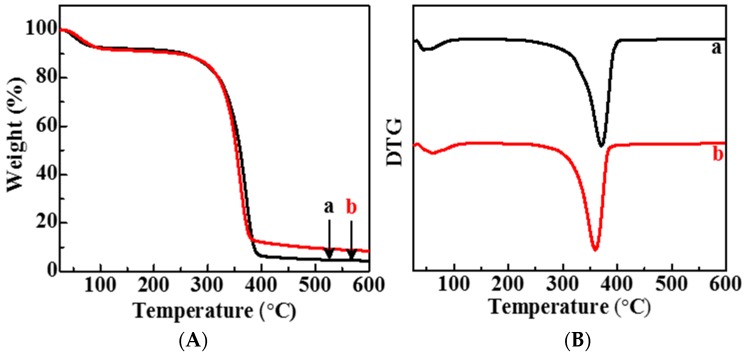
(**A**) TGA and (**B**) DTG analysis of PC (a) and PC_5_ (b) films.

The thermogravimetry was applied to study the weight changes of PC composite film in accordance with increasing temperature, in the form of programmed heating in the range of 25–600 °C under a nitrogen atmosphere. The TG and Differential Thermal Gravity (DTG) spectra of PC and PC_5_ films are shown in Figure 4A,B, respectively. For PC films (Curve a), they are subjected to a slight weight loss before 100 °C, and then a rapid one in the range of 250–400 °C. The first weight loss was mainly caused by the evaporation of physically adsorbed- and hydrogen bond-linked water molecules, and the second one was due to the thermal degradation and decomposition of cellulose and pectin with the generation of C, CO, CO_2_, and H_2_O. The residue of PC film is 4.35%. The maximal decomposition temperature (*T*_max_) of PC film (Curve a) is 370.8 °C, which can be found in Figure 4B. Two weight loss stages were also displayed in the TGA curve of PC_5_ films. The residue of PC_5_ film increased to 8.47% because of the existence of cinnamaldehyde and TP. This result was further confirmed by the shift of the DTG peak from 370.8 to 359.5 °C. Based on these results, the thermal stability slightly decreases after loading with cinnamaldehyde and TP.

### 3.4. Bacterial Inhibition Zones

The disc diffusion method was used to evaluate the bacterial inhibition properties of PC composite films. The bacterial inhibition capacities were estimated by measuring the inhibition zone diameters around the samples after 24 h incubation.

**Figure 5:**
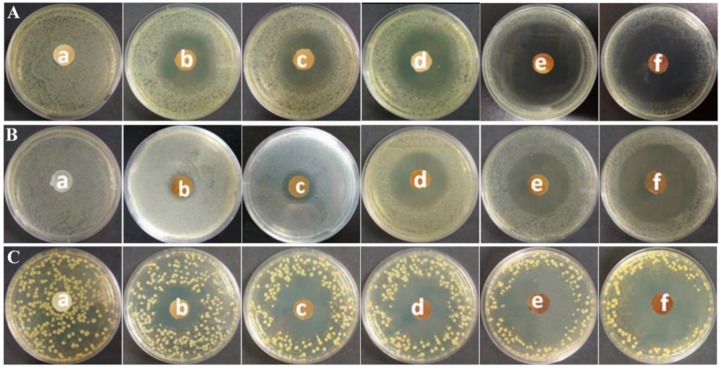
Optical images of inhibition zones: (**A**) *E. coli*; (**B**) *C. albicans*; (**C**) *S. aureus*. (In all plates, a–f are PC, PC_1_, PC_2_, PC_3_, PC_4_, and PC_5_ films, respectively).

**Figure 6:**
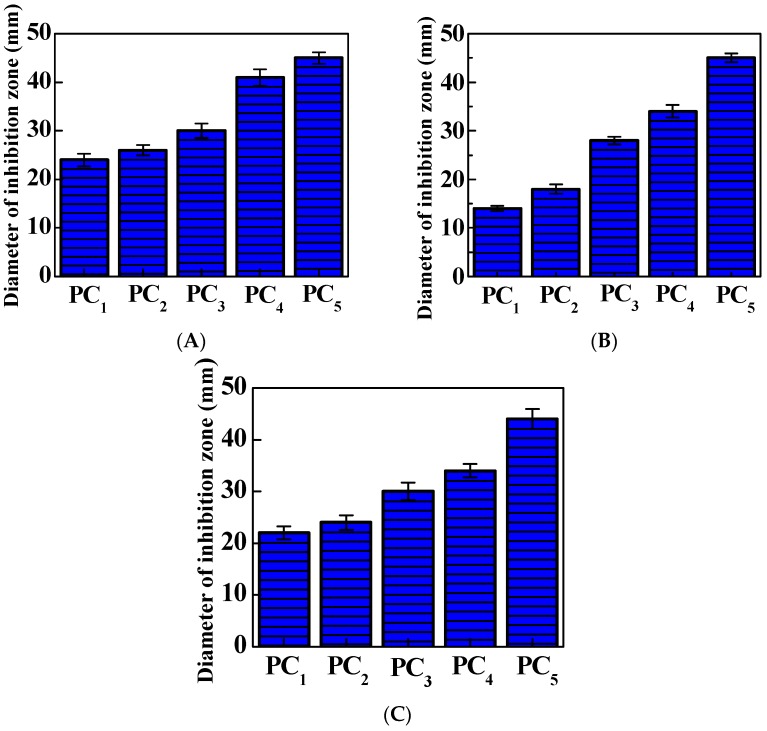
Average diameters of inhibition zones of PC composite films: (**A**) *E. coli*; (**B**) *C. albicans*; (**C**) *S. aureus*.

Figure 5 shows the inhibition zone pictures. For PC films (a), no inhibition zones were observed at all against *E. coli*, *C. albicans*, and *S. aureus*, indicating that PC films do not possess any bacterial or fungal inhibition abilities. However, after loadings of cinnamaldehyde and TP into PC films, they exhibit clear inhibition zones around *E. coli*, *C. albicans*, and *S. aureus*. Meanwhile, the inhibition ability increases with increasing loadings as well. Figure 6 lists the average inhibition zone diameters of PC composite films. PC_1_ films had the smallest diameters of inhibition zones of 24, 14, and 22 mm against *E. coli*, *C. albicans*, and, *S. aureus*, respectively. Meanwhile, PC_5_ films exhibited the best bacterial and fungal inhibition activities, with inhibition zone diameters of 45, 45, and 44 mm against *E. coli*, *C. albicans*, and, *S. aureus*, respectively. It can be clearly seen that the inhibition zone increases with the increase of cinnamaldehyde and TP loadings in the PC composite films. The present study clearly shows that the PC composite films show excellent bacterial and fungal inhibition activities.

### 3.5. Antioxidant Properties

**Figure 7:**
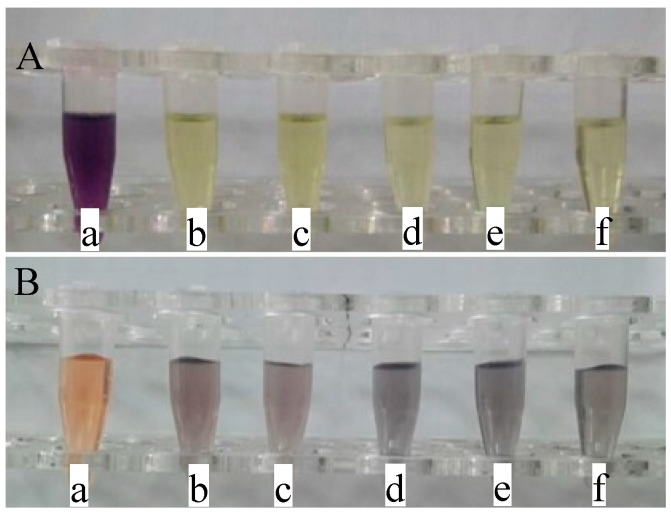
Pictures of color changes of DPPH and hydroxyl free radicals after being treated with PC composite films (**A**) DPPH. (**B**) hydroxyl free radicals.

**Figure 8:**
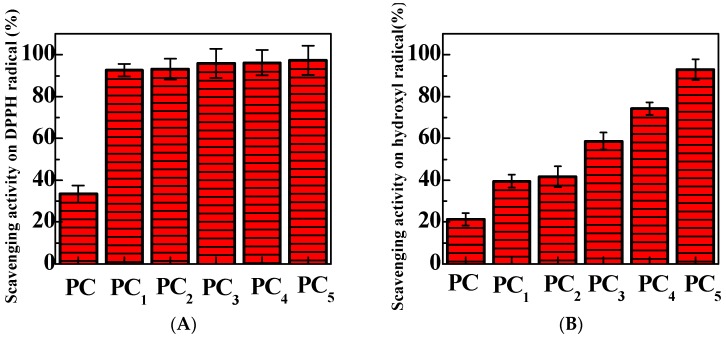
Average scavenging effects of PC composite films on (**A**) DPPH and (**B**) hydroxyl free radicals.

DPPH is a stable free radical, and it is usually used for the identification of antioxidant properties [19]. In this study, DPPH radical scavenging assays were performed to evaluate the antioxidant activities of PC composite films. The solution color changes are presented in Figure 7. DPPH is a relatively stable radical which can be reduced to the corresponding hydrazine easily [20]. DPPH was dissolved in ethanol, and showed a deep purple solution. Sample a in Figure 7A is the color of the DPPH solution after treatment with PC films, which exhibits a deep purple. As shown in Figure 7A, the color of the DPPH solution (Sample b) became yellow after treatment with PC_1_ composite films, indicating that PC_1_ composite films exhibit some antioxidant properties. With the loading increase of TP in the PC composite films, the color of the DPPH solutions decreased. For PC_5_ composite films (Sample f), the DPPH solution became light yellow, indicating the good antioxidant ability of PC_5_ composite films. The DPPH free radical scavenging percentages are shown in Figure 8A. PC composite films without any TP loading have a DPPH free radical scavenging percentage of 33%. Meanwhile, PC_1_, PC_2_, PC_3_, PC_4_, and PC_5_ composite films exhibited enhanced DPPH free radical scavenging abilities. With the loading increase of TP in the PC composite films, the scavenging percentages of DPPH free radicals increased from 92% to 97%. This result is constant with the solution color changes shown in Figure 7A.

Figure 7B shows the solution colors of the tested system after treatment with PC, PC_1_, PC_2_, PC_3_, PC_4_, and PC_5_ composite films. The 1,10-phenanthroline-FeSO_4_-H_2_O_2_ system exhibits an orange color. For PC films (Sample a), the solution still shows an orange color. However, the solution color changed to grey after being treated with PC_1_ composite films (Sample b). The color became dark grey with the increasing TP loadings in the PC composite films (Samples c–f). The scavenging abilities of PC composite films for hydroxyl free radicals are shown Figure 8B. The scavenging abilities of PC composite films for hydroxyl radicals increase significantly as the TP loading increases. Meanwhile, the antioxidant capacities of PC composite films exhibited a TP dose-dependent scavenging activity. The scavenging effect of PC composite films with any TP loading in the PC composite film for hydroxyl radicals is 21%. Whereas, the hydroxyl free radical scavenging percentage for the solution treated with PC_1_ composite film is 40%. This indicates that PC_1_ composite films have an increased scavenging ability for free radicals. Moreover, the scavenging activity for hydroxyl radicals increased with increasing TP loadings in the PC composite films. For PC_5_ composite films, the scavenging percentage for hydroxyl free radicals reached 93%.

Above all, PC films exhibit some DPPH radical and hydroxyl radical scavenging activity. This slight antioxidant activity may be due to the supply of hydrogen by polysaccharides including cellulose and pectin [21]. PC_5_ composite films have the best scavenging ability against both DPPH free radicals and hydroxyl radicals. The great antioxidant performance is due to the existence of TP in the PC composite films, which can interrupt the chain oxidation reactions by donating a hydrogen atom, acting as an acceptor of free radicals, or by chelating metals [22].

### 3.6. Mechanical Properties

**Figure 9:**
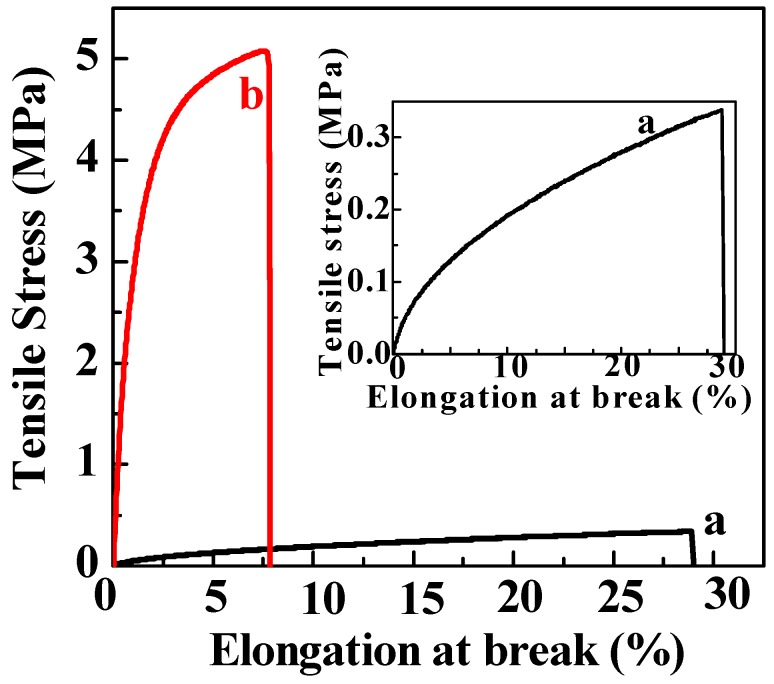
Typical stress—strain curves of PC (a) and PC_5_ (b) films.

In order to ensure that the PC-based films have good mechanical properties which can be applied for food packaging, tensile stress tests were carried out. The tensile stress, elongation at break, and thickness of PC and PC_5_ films are summarized in Table 2. The stress-strain curves are shown in Figure 9, and the inset image is the enlarged curve of PC film. As shown in Table 2, the tensile strengths of PC and PC_5_ films were 0.31 and 5.18 MPa, respectively. The tensile strength was highly improved with the incorporation of cinnamaldehyde and TP, which could act as cross-linkers of PC films. Additionally, the elongation at the break decreased from 28.1% for PC films to 7.4% for PC_5_ films. Therefore, loading cinnamaldehyde and TP into PC films enhances their mechanical properties, making them more suitable for the application of food packaging.

## 4. Conclusions

The study explored the effects of integration of TP and cinnamaldehyde on the antibacterial and antioxidant properties of biodegradable PC composite films. TG curves showed that the whole thermal stability of PC composite film was slightly decreased with the loading of TP and cinnamaldehyde. The prepared PC composite films displayed a significant inhibitory effect against *E. coli*, *C. albicans*, and *S. aureus*. Meanwhile, they also exhibited good scavenging effects on DPPH and hydroxyl free radicals. Based on the obtained results, the prepared PC composite films could be considered as a potential candidate in the food packaging and preservation field.

## Figures and Tables

**Table 1:** Cinnamaldehyde and tea polyphenols concentrations in the PC films.

	Cinnamaldehyde (%)	TP Concentration (mg/mL)
PC_1_	1	0.2
PC_2_	2	0.4
PC_3_	3	0.6
PC_4_	4	0.8
PC_5_	5	1

**Table 2:** Tensile testing results of the PC and PC_5_ films.

	Tensile Strength (MPa)	Elongation at Break (%)	Thickness (μm)
**PC film**	0.31 ± 0.03	28.1 ± 1.9	504 ± 6
**PC_5_ film**	5.18 ± 0.10	7.4 ± 0.4	345 ± 9

## References

[1] Yu Z., Li B., Chu J., Zhang P. (2018). Silica in situ enhanced PVA/chitosan biodegradable films for food packages. Carbohydr. Polym..

[2] Wu Z., Huang X., Li Y.C., Xiao H., Wang X. (2018). Novel chitosan films with laponite immobilized Ag nanoparticles for active food packaging. Carbohydr. Polym..

[3] Gregorova A., Saha N., Kitano T., Saha P. (2015). Hydrothermal effect and mechanical stress properties of carboxymethylcellulose based hydrogel food packaging. Carbohydr. Polym..

[4] Shankar S., Tanomrod N., Rawdkuen S., Rhim J.W. (2016). Preparation of pectin/silver nanoparticles composite films with UV-light barrier and properties. Int. J. Biol. Macromol..

[5] Farahnaky A., Sharifi S., Imani B., Dorodmand M.M., Majzoobi M. (2018). Physicochemical and mechanical properties of pectin-carbon nanotubes films produced by chemical bonding. Food Packag. Shelf Life.

[6] Pandele A.M., Andronescu C., Vasile E., Radu I.C., Stanescu P., Iovu H. (2017). Non-covalent functionalization of GO for improved mechanical performances of pectin composite films. Compos. Part A Appl. Sci. Manuf..

[7] Bermudez-Oria A., Rodriguez-Gutierrez G., Vioque B., Rubio-Senent F., Fernandez-Bolanos J. (2017). Physical and functional properties of pectin-fish gelatin films containing the olive phenols hydroxytyrosol and 3,4-dihydroxyphenylglycol. Carbohydr. Polym..

[8] Rezvanian M., Ahmad N., Mohd Amin M.C., Ng S.F. (2017). Optimization, characterization, and in vitro assessment of alginate-pectin ionic cross-linked hydrogel film for wound dressing applications. Int. J. Biol. Macromol..

[9] Chiarappa G., De’Nobili M.D., Rojas A.M., Abrami M., Lapasin R., Grassi G., Ferreira J.A., Gudiño E., de Oliveira P., Grassi M. (2018). Mathematical modeling of L-(+)-ascorbic acid delivery from pectin films (packaging) to agar hydrogels (food). J. Food Eng..

[10] Hennink W.E., van Nostrum C.F. (2012). Novel crosslinking methods to design hydrogels. Adv. Drug Deliv. Rev..

[11] Tummalapalli M., Berthet M., Verrier B., Deopura B.L., Alam M.S., Gupta B. (2016). Composite wound dressings of pectin and gelatin with aloe vera and curcumin as bioactive agents. Int. J. Biol. Macromol..

[12] Nesic A., Onjia A., Davidovic S., Dimitrijevic S., Errico M.E., Santagata G., Malinconico M. (2017). Design of pectin-sodium alginate based films for potential healthcare application: Study of chemico-physical interactions between the components of films and assessment of their antimicrobial activity. Carbohydr. Polym..

[13] Marquez G.R., Di Pierro P., Mariniello L., Esposito M., Giosafatto C.V.L., Porta R. (2017). Fresh-cut fruit and vegetable coatings by transglutaminase-crosslinked whey protein/pectin edible films. LWT-Food Sci. Technol..

[14] Seslija S., Nesic A., Ruzic J., Krusic M.K., Velickovic S., Avolio R., Santagata G., Malinconico M. (2018). Edible blend films of pectin and poly(ethylene glycol): Preparation and physico-chemical evaluation. Food Hydrocoll..

[15] Ye S., He S., Su C., Jiang L., Wen Y.Y., Zhu Z.J., Shao W. (2018). Morphological, Release and Antibacterial Performances of Amoxicillin-Loaded Cellulose Aerogels. Molecules.

[16] Lee J.H., Hwang C.E., Son K.S., Cho K.M. (2019). Comparisons of nutritional constituents in soybeans during solid state fermentation times and screening for their glucosidase enzymes and antioxidant properties. Food Chem..

[17] Feng M., Yu L., Zhu P., Zhou X., Liu H., Yang Y., Zhou J., Gao C., Bao X., Chen P. (2018). Development and preparation of active starch films carrying tea polyphenol. Carbohydr. Polym..

[18] Muhoza B., Xia S., Cai J., Zhang X., Duhoranimana E., Su J. (2019). Gelatin and pectin complex coacervates as carriers for cinnamaldehyde: Effect of pectin esterification degree on coacervate formation, and enhanced thermal stability. Food Hydrocoll..

[19] Zhang D.-Y., Wan Y., Hao J.-Y., Hu R.-Z., Chen C., Yao X.-H., Zhao W.-G., Liu Z.-Y., Li L. (2018). Evaluation of the alkaloid, polyphenols, and antioxidant contents of various mulberry cultivars from different planting areas in eastern China. Ind. Crops Prod..

[20] Jia Y., He Y., Lu F. (2018). The structure-antioxidant activity relationship of dehydrodiferulates. Food Chem..

[21] Luo Q.L., Tang Z.H., Zhang X.F., Zhong Y.H., Yao S.Z., Wang L.S., Lin C.W., Luo X. (2016). Chemical properties and antioxidant activity of a water-soluble polysaccharide from Dendrobium officinale. Int. J. Biol. Macromol..

[22] Dou L., Li B., Zhang K., Chu X., Hou H. (2018). Physical properties and antioxidant activity of gelatin-sodium alginate edible films with tea polyphenols. Int. J. Biol. Macromol..

